# Pentraxin 3 and *Shigella* LPS and IpaB Antibodies Interplay to Defeat Shigellosis

**DOI:** 10.3390/jcm11154384

**Published:** 2022-07-28

**Authors:** Shiri Meron-Sudai, Arava Reizis, Sophy Goren, Anya Bialik, Amit Hochberg, Dani Cohen

**Affiliations:** 1 School of Public Health, Sackler Faculty of Medicine, Tel Aviv University, Tel Aviv 69978, Israel; meronshi@tauex.tau.ac.il (S.M.-S.); aravareizis@gmail.com (A.R.); sophyg@tauex.tau.ac.il (S.G.); bialika@tauex.tau.ac.il (A.B.); 2 Newborn and Neonatal Care Department, Hillel Yaffe Medical Center, Hadera 38100, Israel; amit.hochberg@gmail.com

**Keywords:** *Shigella*, pentraxin 3 (PTX3), c-reactive protein (CRP), Lipopolisaccharide (LPS), invasion plasmid antigen B (IpaB)

## Abstract

*Shigella* causes moderate to severe diarrhea or dysentery after invading the colon mucosa. Long Pentraxin 3 (PTX3) is recognized as the humoral component of the innate immune response to bacterial pathogens. We examined the interplay between levels of PTX3 and levels of anti-*Shigella* lipopolysaccharide (LPS) and anti-*Shigella* type 3 secretion system protein-IpaB antibodies in children during acute shigellosis and after recovery. PTX3 concentrations in serum and stool extracts were determined by sandwich ELISA using commercial anti-PTX3 antibodies. Serum IgG, IgM, and IgA anti-*S. sonnei* LPS or anti-*S. sonnei* IpaB were measured using in house ELISA. Children with acute shigellosis (*n* = 60) had elevated PTX3 levels in serum and stools as compared with recovered subjects (9.6 ng/mL versus 4.7 ng/mL, *p* < 0.009 in serum and 16.3 ng/g versus 1.1 ng/g in stool, *p* = 0.011). Very low levels of PTX3 were detected in stools of healthy children (0.3 ng/g). Increased serum levels of PTX3 correlated with high fever accompanied by bloody or numerous diarrheal stools characteristic of more severe shigellosis while short pentraxin; C-Reactive Protein (CRP) did not show such a correlation. PTX3 decreased in convalescence while anti-*Shigella* antibodies increased, switching the response from innate to adaptive toward the eradication of the invasive organism. These data can inform the development of *Shigella* vaccines and treatment options.

## 1. Introduction

*Shigella* is a leading cause of moderate to severe diarrhea among children worldwide. More than 250 million cases of shigellosis are estimated to occur annually and over 63,000 deaths occur among children under 5 years of age in low-and middle-income countries [1]. Shigellosis is also common in high-income countries, occurring mostly among toddlers living in crowded communities and day-care centers [2]. *Shigella* is a gram-negative bacterium belonging to the enterobacteriaceae family and genetically closely related to *E. coli*. There are four species of *Shigella*: *S. dysenteriae*, *S. flexneri*, *S. boydii*, and *S. sonnei*, where *S. flexneri* accounts for most cases in low-and middle-income countries and *S. sonnei* accounts for most cases in high-income countries [3,4]. Invasion of *Shigella* to the colonic mucosa triggers an innate immunity-related inflammatory response [5,6,7]. Pentraxins are a super family of proteins divided as short and long pentraxins according to their structure. C-reactive protein (CRP) is a prototype of short pentraxins produced in the liver due to stimulation with IL-6. CRP levels in blood serve as a marker for suspected inflammation. On the other hand, Pentraxin 3 (PTX3) is a prototype of long pentraxin, first to be identified during the 1990s [8,9,10]. It is secreted by mononuclear phagocytes, dendritic cells, and neutrophils and is a key component of the humoral innate response to bacterial pathogens [11]. In addition to being produced by components of the innate immune system, PTX3 is also secreted in response to pro-inflammatory signals by different cell types such as vascular cells, fibroblasts, smooth muscle cells, glial cells, and chondrocytes [12,13]. Unlike CRP, which is produced in response to IL-6 stimulation, PTX3 secretion is induced in response to IL-1β and TNF-α [9]. PTX3 has a dominant role in trauma and inflammation, being able to interact with variable ligands such as growth factors, components of the complement system, and extracellular matrix components [13,14]. It also serves as a humoral pattern recognition receptor (PRR) capable of binding different pathogens including *Staphylococcus aureus*, *Pseudomonas aeruginosa*, *Nisseria meningitides*, *Streptococcus pneomoniae*, and *Aspergillus fumigatus* [12,14]. PTX3 is also involved in *Shigella* pathogenesis. Both *S. flexneri* and *S. sonnei* triggered secretion of PTX3 by bone marrow-derived dendritic cells and by monocytes-derived dendritic cells [15]. PTX3-bound *S. flexneri* bacteria had a reduced ability to invade epithelial cells, were better killed by complement, and more efficiently internalized by macrophages [15]. In that particular study, we showed that levels of PTX3 in plasma from *S. sonnei* shigellosis patients were significantly higher than in a healthy control group and that PTX3 plasma levels in shigellosis patients were associated with symptom severity [15]. In the present study we quantified the presence of PTX3 in serum and stool samples of cases of culture proven shigellosis and studied the interplay between levels of PTX3 as a component of the innate immune response and the levels of anti-*Shigella* lipopolysaccharide (LPS) and anti-*Shigella* type 3 secretion system protein-Invasion plasmid antigen B (IpaB) antibodies in acute and convalescent phases of shigellosis. We also examined the association between disease-related characteristics and PTX3 as a locally released inflammatory marker in comparison to CRP, which represents a systemic inflammatory indicator.

## 2. Materials and Methods

### 2.1. Study Population

Serum samples were collected from 44 children (mean age: 4.8 ± 2.2 years, range: 6 months—10 years; 45% females) at the acute phase of culture proven shigellosis (1–7 days after hospital or clinic admission) and from 20 children (mean age: 5.8 ± 3.2 years, range: 2.5–15 years; 26% females) at the convalescent phase (8 days–6 months after hospital or clinic admission) of shigellosis. Stool samples were obtained from 16 children (mean age: 2.9 ± 1.5 years, range: 3 months–4.7 years; 31% females) at the acute phase of disease, 12 children (mean age: 4.8 ± 1.8 years, range: 2–8.5 years; 27% females) at the convalescent phase and from 29 control children without diarrhea or any infectious disease diagnosis (mean age: 1.6 ± 1.3 years, range: 1.2 months–5 years; 32% females). Subjects were recruited from several medical centers and community clinics in Israel. A clinical evaluation form was filled out by a physician recording symptoms and clinical parameters. Consent forms were signed by all children’s parents and by adult participants. The study was approved by the Helsinki Committee of Hillel Yaffe Medical Center and by the Israel Ministry of Health Ethics Committee (AH-382-11).

### 2.2. Serum and Stool Collection

Serum and stool samples were received from hospitals and clinics and stored at −80 °C until assayed.

### 2.3. PTX3 Extraction from Stool Samples

Cryopreserved stool samples (approximately 100–400 mg) were mixed with Dulbecco’s Phosphate Buffered Saline (DPBS), 1 g/2 mL by aggressive vortex. Tubes were left to stand at room temperature for 2 h, with repeated vortex every 10–20 min. Samples were centrifuged at 12,000× *g* in 4 °C for 30 min and the upper supernatant was collected and transferred to a new tube. Samples were stored at −80 °C until assayed.

### 2.4. PTX3 Measurement

PTX3 concentrations in serum and stool extracts were determined by sandwich ELISA and according to the manufacturer’s protocol (DuoSet, DY1826, R&D systems, Minneapolis, MN, USA). Briefly, 96-well microplates (Corning-Costar Inc., Raleigh, NC, USA) were coated overnight with coating antibody, washed using automated washer (Thermo Scientific, Waltham, MA, USA), and blocked for 1.5 h with 1% Bovine Serum Albumin (BSA) (EMD Millipore Corp, Burlington, MA, USA) in DPBS. Plates were washed and serum samples diluted 1:5 or stool extracts diluted 1:2 in reagent diluent containing 10% Fetal Bovine Serum (FBS) (Rhenium) were added and incubated for 2 h; plates were washed and incubated with detection antibody for 2 h. After washing, 100 µL/well of Streptavidin-HRP (R&D systems) was added for 20 min, washed and plates were incubated with 3,3′,5,5′-Tetramethylbenzidine (TMB) substrate (EMD Millipore Corp) for 20 min. The reaction was stopped by adding 2N H_2_SO_4_ (Merck, Kenilworth, NJ, USA). Optical density (OD) was measured at 450 nm–570 nm using an ELISA plate reader (Thermo Scientific Multiskan FC; Waltham, MA, USA). PTX3 concentrations measured in stool extracts were further calculated for 1 g stool.

### 2.5. Serum Antibodies Measurement

Levels of serum IgG or IgM or IgA anti-*S. sonnei* LPS or anti-*S. sonnei* IpaB were measured using in house ELISA. Then, 96-well microtiter plates (Corning-Costar Inc.) were coated with 10 µg/mL *S. sonnei* LPS or 0.1 µg/mL IpaB (kindly provided by Prof. Wendy L. Picking, Oklahoma State University, Stillwater, OK, USA) in 0.05 M Carbonate Buffer (PH 9.6). Unbound sites were blocked with blocking buffer containing 0.5% BSA (EMD Millipore Corp) and 0.5% Casein (Merck) for 1 h at 37 °C. Plates were washed and serum samples 2-fold serial diluted (8 dilutions) in blocking buffer were added to the coated wells (initial dilution 1:100), incubated overnight, and washed. Alkaline phosphatase conjugate antibody anti-human IgG or IgM or IgA (KPL, Sera Care, MA, USA) were added and incubated overnight followed by washing. Phosphatase substrate para-Nitrophenylphosphate-pNPP One component Microwell Substrate Solution (Southern Biotech, Birmingham, AL, USA) was added for 15 min and color development was stopped by the addition of 3 M Sodium Hydroxide (Merck). OD was measured at 405 nm using an ELISA plate reader (Thermo Scientific Multiskan FC; Waltham, MA, USA). OD values were corrected by subtraction of OD value of blank wells. Results were expressed as geometric mean of ELISA Units (GMEU) (OD × dilution factor).

### 2.6. Statistical Analysis

Data were analyzed using SPSS version 28 (IBM, New York, NY, USA).

All tests were two tailed and *p* < 0.05 was considered significant. Anti-*Shigella* LPS and IpaB antibody concentrations are expressed as GMEU with 95% confidence intervals (CI). Other continuous data are reported as mean and standard deviation unless otherwise stated. Counts and percentages were employed for categorical variables. Independent samples *t*-test and one-way analysis of variance (ANOVA) were used to test statistical significance of differences in continuous variables (ex. PTX3 mean and antibody GMEU) while chi square or Fisher’s exact test were employed to assess differences in categorical variables (ex. percent with PTX3 above a cutoff).

## 3. Results

### 3.1. Study Groups and Levels of PTX3 and Antibodies to S. sonnei LPS and IpaB Antigens in Serum Samples

The mean of PTX3 levels in serum samples of acute patients was significantly higher than in convalescent patients (9.6 ng/mL vs. 4.7 ng/mL, *p* = 0.009, Table 1). While in the convalescent phase serum level of PTX3 was much reduced compared to the acute phase, anti-LPS and anti-IpaB antibody levels were increased following an inverse trend (Table 1). The GMEU levels of IgG, IgA, and IgM anti- *S. sonnei* LPS as well as IgG anti-IpaB at the convalescent phase were at least 4 times higher compared with GMEU levels in children at the acute phase (*p* < 0.001). A more moderate increase was shown for IgA and IgM anti-IpaB (3.7 and 1.6 increase, respectively), although statistically significant (*p* < 0.001, *p* = 0.019). The elevated levels of antibodies in the convalescent patients were *S. sonnei* specific; no difference in GMEU levels of IgG, IgA, and IgM anti-*S. flexneri* 2a LPS (heterologous antigen) was observed between acute and convalescent patients (data not shown).

### 3.2. PTX3 Levels in Serum Samples Correlated with High Fever Accompanied by Bloody or Numerous Diarrheal Stools Characteristic of More Severe Shigellosis

We examined whether serum levels of PTX3 are positively associated with symptoms or signs of shigellosis (Table 2). Four parameters were available: maximal measured body temperature, number of vomiting episodes, blood in stool, and number of watery stools per 24 h. Among acute patients whose maximal measured body temperature was above 39 °C, the PTX3 level was higher than among patients whose maximal measured temperature was equal or below 39 °C (12.3 ng/mL vs. 5.4 ng/mL, *p* = 0.05) and this difference increased when patients with fever above 39 °C had also bloody stools (*p* = 0.003) or more than 9 watery stools per day (*p* = 0.004). Serum levels of short pentraxin CRP, which is routinely used as a systemic inflammation biomarker, and its association to severity of symptoms was also assessed, though did not correlate with the severity of symptoms or signs of disease. PTX3 levels were higher within the first 2 days of hospital or clinic admission than in later days (14.1 ng/mL vs. 7.5 ng/mL, *p* = 0.063). In line with its relatively late secretion, CRP levels did not differ between the first 2 days and the subsequent 2 days of hospital or clinic admission.

### 3.3. PTX3 Levels in Stool Samples of Acute Shigellosis Patients Are Significantly Higher Than in Stool Samples of Convalescent Patients and Healthy Controls

PTX3 levels were significantly higher in stool samples of acute *S. sonnei* shigellosis patients as compared with PTX3 levels measured in stool samples of convalescent *S. sonnei* shigellosis patients and controls (Table 3). Almost 90% of the acute patients had PTX3 levels higher than 2 ng/g while such levels were found in only 8% of the patients at the convalescent phase and in 3% of the controls (*p* < 0.001). Of the 16 cases of acute culture-proven *S. sonnei* shigellosis, 13 (81.2%) measured fever above 39 °C, 5 of 16 (31.2%) reported bloody stools, and 11 (68.7%) had more than 9 diarrheal stools per 24 h at the acute stage.

## 4. Discussion

PTX3 is a key component of the innate immunity arm. Its secretion by immune system cells such as DC and PMN in response to infection as well as its ability to serve as humoral PRR, binding to different pathogens, makes PTX3 a dominant player in innate immunity. The involvement of PTX3 in the pathogenesis of *Shigella* was previously reported in one study [15]. Here, we showed that PTX3 levels in blood are elevated during the acute phase of shigellosis compared to convalescent patients. At the acute phase, the mean level of PTX3 in serum was twice the mean level of patients at the convalescent phase and approximately four times higher than serum PTX3 level reported in healthy children (≤2 ng/mL) [16,17,18]. This is in line with other studies showing increased levels of plasma PTX3 during other local or mucosal infections [19,20]. The association that we found between PTX3 levels in serum and specific symptoms or signs of more severe disease (high fever accompanied by bloody or numerous diarrheal stools) is consistent with shigellosis being an inflammatory infection of the colon mucosa that induces the corresponding innate response involving recruitment of neutrophils, macrophages, and dendritic cells to the site of infection which rapidly secrete PTX3 to fight the invading bacteria. CRP, which is the gold standard for assessing inflammation, was not associated with these disease manifestations. Due to the fact that PTX3 is secreted at the early stages of infection in response to inflammatory signals at the local site of infection (colon mucosa in the case of shigellosis), an increase over basal level can be observed within the first 6–8 h of inflammation [13,21]. In contrast, CRP, which is secreted at a systemic level, reaches its peak level only 36–48 h after initial inflammation. Our findings confirmed that CRP levels did not differ between the first 2 days and after 2 days of hospital or clinic admission.

We postulate that the PTX3 elevated levels at early acute phase highlight the role of PTX3 as humoral component of the innate response (“ante antibody”) in limiting the pathogenic process of shigellosis during the extracellular phase of the infection. This could include PTX3-binding to shigellae that reduces the ability to invade epithelial cells or by mediating complement killing of the invasive *Shigella* [19]. The elevated levels of IgM, IgG, and IgA anti-*Shigella* LPS or IpaB antigens in the convalescent patients compared with that of acute patients, in parallel with the reduced PTX3 levels, support a dominant role played also by PTX3 in bridging between the innate and the adaptive response as proposed by Chorny et al. That study demonstrated that PTX3 activates splenic marginal zone B cells (MZ B cells), leading to production of IgM and IgG antibodies as well as IgG class switch recombination [22]. Neutrophil B helper cells (NBh) situated in splenic peri-marginal zone areas expressed elevated levels of PTX3 in the presence of GM-CSF and *E. coli* LPS and the released PTX3 bound to MZ B cells. It was also shown that in *Ptx3*^−/−^ mice infected with *Streptococcus pneumonia* (SP) there was impaired production of SP-specific IgM and IgG as well as class switching from IgM to IgG [22].

Our data demonstrated that high levels of PTX3 are secreted locally at the site of *Shigella* infection in the gut. We showed for the first time that PTX3 levels in stool are increased intensely during shigellosis, reaching a mean level of 16.3 ng/g stool compared with 1.1 ng/g in convalescent patients and 0.3 ng/g in healthy controls. PTX3 may contribute to the clearance of *Shigella* and tissue repair at the colonic mucosa level in cooperation with systemic transuding and locally produced antibodies to *Shigella* LPS and type 3 secretion system protein-IpaB. The difference in PTX3 levels seen in stool samples between the acute patients and controls suggest that in the case of shigellosis PTX3 in stool is a more specific inflammation marker than PTX3 levels in serum. This is consistent with the potential occurrence of elevated PTX3 levels in blood due to intercurrent systemic infections or non-infectious reasons such as injuries, cardiac diseases, and chronic kidney disease [14]. The limited number of patients for whom stool samples were collected prevented us from examining whether PTX3 levels secreted in the gut are associated with shigellosis severity as seen for PTX3 levels in blood. We can confirm, however, that the significantly increased PTX3 levels were measured in patients with culture-proven *S. sonnei* shigellosis (shedding the pathogen) and suffering from typical shigellosis symptoms or signs of disease. This study was conducted in well-nourished immunocompetent children living in a high-income country. PTX3 levels decreased in convalescence following an opposite dynamic to the adaptive antibody response to *Shigella* antigens. There is a concerted effect of PTX3 as a humoral component of the innate response and the anti-*Shigella* antibody response, which could explain the limited mucosal invasiveness of shigellae without going deeper and turning shigellosis into a systemic disease. It is not known how these two components of the immune response will interplay in young children in low-and middle-income countries and if local inflammation including the PTX3 response will not persist being part of the risk factors for stunting and other long-term negative effects of shigellosis.

## 5. Conclusions

Our data showed that during shigellosis PTX3 is highly secreted systemically as well as locally and the decrease in secretion during convalescence paralleled with the increase in anti-*Shigella* LPS and IpaB antibodies. There is a concerted effect of PTX3 as a humoral component of the innate response and the anti-*Shigella* antibody response in successfully fighting shigellosis.

PTX3 serum levels correlated with specific characteristics of shigellosis severity as opposed to CRP levels, which showed no such correlation, suggesting that PTX3 might be a better indicator of the colon mucosal inflammation. Further studies are needed to confirm or refute this observation.

## Figures and Tables

**Table 1:** Study groups and levels of PTX3 and antibodies to *S. sonnei* LPS and IpaB antigens in serum samples.

Parameter	*n*	Acute Phase	*n*	Convalescent Phase	*p*-Value vs. Acute
PTX3 ng/Ml Mean (95% CI)	44	9.6 (6.2–12.9)	20	4.7 (3.4–6.1)	0.009
IgG anti-LPS GMEU (95% CI)	41	57.8 (41.4–80.5)	17	281.7 (159.3–497.9)	<0.001
IgA anti LPS GMEU (95% CI)	41	10.4 (6.5–16.4)	16	59.1 (24.8–140.7)	<0.001
IgM anti LPS GMEU (95% CI)	41	147.8 (110.8–197.2)	16	590.5 (345.8–1008.4)	<0.001
IgG anti-IpaB GMEU (95% CI)	35	37.4 (26.5–52.7)	16	166.3 (91.2–303.4)	<0.001
IgA anti -IpaB GMEU (95% CI)	35	3.9 (2.7–5.5)	15	14.5 (7.4–28.5)	<0.001
IgM anti-IpaB GMEU (95% CI)	35	80.4 (62.1–104.1)	15	135 (101.4–179.7)	0.019

PTX3: Pentraxin 3; LPS: Lipopolysaccharide; IpaB: Invasion plasmid antigen B; IgG, IgA, IgM: Immunoglobulin G, A, M; GMEU: Geometric Mean of ELISA Units; CI: Confidence Intervals.

**Table 2:** Clinical manifestation of disease, days after onset, and level of PTX3 and CRP in serum samples of acute cases of *S. sonnei* shigellosis.

Severity of Symptoms		*n*	Mean PTX3 ng/mL (95% CI)	*p*-Value	*n*	Mean CRP mg/L (95% CI)	*p*-Value
Maximum temp. at acute stage	up to 39 °C	16	5.4 (2.5–8.3)	0.050	16	84.2 (35.1–133.2)	0.752
	above 39 °C	27	12.3 (7.2–17.3)		24	93.8 (54.1–133.5)	
No. of vomiting episodes	up to 3	19	7.3 (4.4–10.2)	0.305	18	84.1 (42.4–125.7)	0.728
	4+	16	11.1 (3.4–18.8)		15	74.5 (35.7–113.4)	
Blood in stool	no	24	7.8 (4.7–10.9)	0.198	22	81.8 (37.6–126)	0.702
	yes	18	12.3 (4.9–19.7)		17	93.4 (49.6–137.3)	
No. of watery stools per 24 h	up to 8	17	6.9 (4–9.9)	0.194	16	78.5 (42.4–114.7)	0.555
	9+	26	11.5 (6.1–16.9)		24	96.5 (51.3–141.7)	
Temp above 39 °C andblood in stool	no	33	6.8 (4.5–9.1)	0.003	31	88.9 (51.8–126)	0.903
	yes	11	17.8 (6.4–29.2)		10	84.8 (42.9–126.6)	
Temp above 39 °C andNo. of watery stools 9+	no	28	6.0 (4–8)	0.004	27	78.3 (46.4–110.2)	0.362
	yes	16	15.8 (7.6–23.9)		14	106.3 (41.7–170.9)	
Days after hospital or clinic admission	<2 days	15	14.1 (8.1–20.2)	0.063	13	96.6 (26.4–166.8)	0.753
	≥2 days	27	7.5 (3.2–11.8)		27	86.6 (54.7–118.6)	

PTX3: Pentraxin 3; CRP: C-Reactive Protein; CI: Confidence Intervals.

**Table 3:** PTX3 in stool samples of shigellosis patients and healthy controls.

Parameter	N	Acute Phase	*n*	Convalescent Phase	*p*-Value vs. Acute	*n*	Controls (Children)	*p*-Value vs. Controls
PTX3 ng/g stool Mean (95% CI)	16	17.75 (5.3–30.2)	12	1.1 (0–3.5)	0.011	29	0.33 (0–0.7)	0.008
PTX3 > 2 ng/g stool		88%		8%	<0.001		3%	<0.001

PTX3: Pentraxin 3.

## Data Availability

Data presented in this study without any participant personal identifiers will be made available on request from the corresponding author.
